# Suicidal Ideation Is Associated With Reduced Functional Connectivity and White Matter Integrity in Drug-Naïve Patients With Major Depression

**DOI:** 10.3389/fpsyt.2022.838111

**Published:** 2022-03-21

**Authors:** Joana Vanessa Reis, Rita Vieira, Carlos Portugal-Nunes, Ana Coelho, Ricardo Magalhães, Pedro Moreira, Sónia Ferreira, Maria Picó-Pérez, Nuno Sousa, Nuno Dias, João M. Bessa

**Affiliations:** ^1^Life and Health Sciences Research Institute, School of Medicine, Braga, Portugal; ^2^ICVS/3B's—PT Government Associate Laboratory, Braga/Guimarães, Portugal; ^3^2CA-Braga, Clinical Academic Center, Hospital de Braga, Braga, Portugal; ^4^Psychological Neuroscience Lab, Centro de Investigação em Psicologia (CIPsi), School of Psychology, University of Minho, Braga, Portugal; ^5^2Ai—School of Technology, Instituto Politécnico do Cávado e do Ave (IPCA), Barcelos, Portugal

**Keywords:** major depression, suicide, suicidal ideation, fMRI, DTI

## Abstract

Depression is a highly prevalent psychiatric disorder affecting millions of people worldwide. Depression is characterized by decreased mood or loss of interest in daily activities, changes in feeding and circadian rhythms and significant impairments in cognitive and executive function. In addition, the occurrence of recurrent thoughts of death and suicidal ideation confers depressed patients a higher risk of suicide than the general population. With this study, we aimed to explore the neural correlates of suicidal ideation in drug-naïve patients diagnosed with depression. Twenty-five patients were scanned using two-different magnetic resonance imaging (MRI) modalities, resting state functional MRI (fMRI) and diffusion tensor imaging (DTI). Resting state allowed the exploration of connectivity patterns in the absence of a specific stimulus and DTI allowed a detailed analysis of structural white matter integrity with measures like fractional anisotropy (FA). Probabilistic independent component analysis (PICA), network-based statistics and tract-based spatial statistics (TBSS) were applied to analyze resting-state fMRI and DTI data, respectively. Our results showed that, in our sample of drug-naïve patients, suicidal ideation was negatively associated with resting-state functional connectivity in the visual networks and with FA in the genu of corpus callosum and in the right anterior corona radiata. In addition, a significant association was identified between suicidal ideation and a functional connectivity network that included connections between regions in the superior and orbitofrontal cortex, the cerebellum, the cingulate gyrus as well as temporal and occipital regions. In conclusion, this work has expanded our knowledge about the possible functional and structural neuronal correlates of suicidal ideation in drug-naïve patients with depression, paving the way for future personalized therapeutic approaches.

## Introduction

Depression affected nearly 300 million people around the world in 2019 (1) and it is considered by the world health organization (WHO) as the single largest contributor for disability worldwide (2). Nowadays, its prevalence might be even higher due to the challenges imposed by the COVID-19 pandemic (3, 4). The symptoms of depression include depressed mood or loss of interest in daily activities but also, changes in body weight, sleep disturbances, psychomotor retardation, fatigue, feelings of worthlessness, diminished ability to concentrate, recurrent thoughts of death and suicidal ideation (5). In fact, depression is one of the most common diagnosis at the time of death by suicide (6, 7) being depressed patients 20 times more likely to commit suicide than the general population (8, 9). It has been reported that more than 700,000 deaths are a result of suicide (10, 11) and it is estimated that for each death by suicide, there is a much higher number of suicidal attempts, increasing the personal, social, and economic burden of the disorder (8, 12).

Suicide is a complex phenomenon in which many factors could be implicated, especially in MDD patients. Even though the specific pathophysiological pathways of suicide are not yet fully understood, recent research suggest that suicide is a result of not a single factor but rather a complex interaction between genetic vulnerability as well as exogenous and endogenous stressors (13, 14).

The first step on this complex pathway is suicidal ideation, reported as one of the major risk factors for suicide (15). Its presence as a symptom of depression was previously associated with poorer treatment response (16) and deficits in cognitive functions, such as executive functions (17, 18) and verbal learning (19). One third of the patients experiencing suicidal ideation will act on their thoughts (20). Hence, suicidal ideation is a red flag for clinical concern, and it is an important part of the suicide risk assessment guidelines (21).

In the past decade, there has been an exponential growth of studies employing neuroimaging techniques to investigate structural, functional, and metabolic brain processes related to suicidal thoughts and behaviors (22). A commonly used neuroimaging technique is magnetic resonance imaging (MRI), given that its different modalities, such as structural MRI, diffusion tensor imaging (DTI) and functional MRI (fMRI), allow a wide characterization of the brain. Specifically, they provide information on gray and white matter anatomy, structure, as well as brain activity and connectivity. In the literature, these MRI modalities have been used to explore the neural correlates of suicidal ideation due to its importance on the suicide pathway.

At the functional level, previous studies have described the functional networks associated with suicidal ideation in depressed patients, showing a relation between decreased functional connectivity with suicidal ideation (23, 24). The functional networks included similar frontal brain regions, such as the orbitofrontal gyrus, but also distinct regions like the middle occipital gyrus, superior parietal gyrus, thalamus, and caudate (23, 24). Other studies, using seed-based approaches reported increased resting-state functional connectivity (rsFC) between both sides of hippocampus (23), amygdala-precuneus/cuneus (25) and between the left habenula and the left cerebellum (26) when comparing depressed patients with and without suicidal ideation. In contrast, decreased rsFC between the right anterior cingulate cortex and the right middle temporal pole and the orbitomedial prefrontal cortex (27), as well as between the right habenula and the right precuneus and the left inferior frontal gyrus (26) were also described when comparing the same groups of patients with depression.

Alterations in gray and white matter have been reported in depressed patients with suicidal ideation (23, 28, 29). In particular, patients with suicidal ideation showed decreased cortical thickness in the left hemisphere, mainly in the insula when compared to patients without suicidal ideation (29); however, there were no statistically significant differences in their volumes (29). The authors also found that depressed patients with suicidal ideation presented increased radial diffusivity (RD) and decreased fractional anisotropy (FA) in the hippocampal part of the cingulum buddle, superior and posterior corona radiata (CR) as well as anterior thalamic radiation (ATR), when compared to depressed controls (29). These findings suggest a disruption in the white matter integrity of bundles described above, responsible to connect frontal brain regions and basal ganglia (29). Another study also reported decreased white matter integrity in depressed patients with suicidal ideation in tracts connecting frontal regions, namely corpus callosum (30). Indeed, Myung and collaborators (28) also identified a brain network with decreased structural connectivity in frontal, parietal, occipital, and subcortical regions (i.e., thalamus, caudate and pallidum).

These findings suggest that alterations in structure and function of frontal-subcortical networks are implied in suicidal ideation in MDD. Nevertheless, more studies are required in order to consolidate these results and establish the brain patterns related to suicidal ideation. Thus, combining multimodal MRI techniques seems crucial to level up the characterization of the pathophysiology and etiology of suicidal ideation in MDD, which may lead to the development of useful approaches to identify high-risk patients and targeted treatments for suicidal ideation.

In the present study, our main objective was to explore the neural correlates of suicidal ideation in drug-naïve depressed patients at the whole-brain level, using two different MRI modalities, namely resting-state fMRI, and DTI. For that purpose, probabilistic independent component analysis (PICA), network-based statistics (NBS) and tract-based spatial statistics (TBSS) were applied to analyze resting-state fMRI and DTI data, respectively. Our main hypothesis was that suicidal ideation would be associated with decreased functional connectivity as well as white matter integrity.

## Methodology

### Ethics Statement

The study was conducted in accordance with the Declaration of Helsinki (59th Amendment) and was approved by relevant local ethics review boards from University of Minho and Hospital de Braga (Braga, Portugal). The study goals, as well as the psychological and clinical assessments were explained to the participants, of whom all gave informed consent. The anonymity and confidentiality of the participants in the study were guaranteed during all research activities.

### Characterization of Participants

A sample of treatment-naïve (first depressive episode and without prior exposure to antidepressant treatment) MDD patients were recruited in the context of emergency psychiatric evaluations or the outpatient unit of the Psychiatry Department of the Hospital de Braga. The recruitment took place between January of 2016 and January of 2020. The diagnosis was confirmed with the SCID (structured clinical interview for DSM disorders) according to DSM-IV-TR (31).

The inclusion criteria were; being drug-naive, first-episode MDD patient, aged 18-65 years-old, without psychiatric or neurological comorbidities, The exclusion criteria were: age inferior to 18 years or superior to 65 years, presenting any MRI contradictions, comorbid psychiatric disorders (e.g., bipolar disorder, addictive disorders and schizophrenia), prior medical history of neurological disorders or traumatic brain injury and sign of cognitive impairment defined by scores below or equal to 24 in the Mini-Mental Sate Examination (MMSE) test (32).

In accordance with the established criteria, 32 patients were first selected. From these, 7 were not included in this analysis because either they were unable to perform the MRI, or they had to be excluded due to brain abnormalities. Therefore, a total of 25 MDD subjects (8 males, 17 females) were included in the final analysis of resting-state MRI data and 23 MDD subjects (7 males, 16 females) were included in the final analysis of diffusion data (2 subjects did not perform diffusion acquisitions) (Supplementary Figure 1).

All participants performed a psychological assessment in the same day of the MRI acquisition. 17-item Hamilton Depression Rating Scale HAM-D (33) and 14-item Hamilton Anxiety Rating Scale HAM-A (34) were used for the assessment of depression and anxiety severity in the past week, respectively. Both scales are clinician-administered. 10-item Perceived Stress Scale PSS-10 (35) was used to evaluate the global perceived stress in the past month. Beck Scale for Suicidal Ideation BSSI (36) is a self-reported scale used to detect and measure the severity of current suicidal ideation. It comprises 21 items: the first 19 items focus on several characteristics of suicidal ideation; the last two items assess the number of previous suicide attempts and the intention to die associated with it. Each item is rated on a 3-point scale (0–2). The total score is computed by adding the first 19 items of the scale, ranging from 0 to 38. There are no specific cut-off scores; higher total scores reflect higher severity of suicidal ideation.

### MRI Acquisitions

MRI acquisitions were performed at Hospital de Braga with a clinical approved Siemens Magnetom Avanto 1.5 T (Siemens Medical Solutions, Erlangen, Germany) using a 12-channel receive-only head coil. All patients underwent the same MRI acquisition protocol. Even though the protocol included other acquisitions, for the present study only the resting-state functional images and the diffusion-weighted imaging (DWI) sequences were considered.

Functional images were acquired using a BOLD sensitive echo-planar imaging (EPI) with the following parameters: 30 interleaved axial slices, repetition time (TR) = 2,000 ms, echo time (TE) = 50 ms, flip angle (FA) = 90°, slice thickness = 3.5 mm, slice gap = 0.48 mm, in-plane resolution = 3.5 × 3.5 mm^2^, field of view (FoV) = 224 mm and 190 volumes. Also, a T1-weighted structural image for anatomical reference was obtained using a magnetization-prepared rapid acquisition (MPRAGE) by gradient echo sequence with voxel resolution 1.0 × 1.0 × 1.0 mm, FoV 234 × 234 mm^2^, FA of 7°, 176 slices and TE/TR of 3.48/2,730 ms. During functional resting state acquisitions, participants were instructed to remain in an awake and calm state, with their eyes closed while trying not to focus on any particular thought.

DWI scans were performed using a spin echo–echo planar imaging (SE-EPI) sequence: TR = 8,800 ms, TE = 99 ms, FoV = 240 mm × 240 mm, acquisition matrix = 120 × 120, 61 two-millimeter axial slices with no gap, 30 non-collinear gradient directions with *b* = 1,000 s mm^−2^, one *b* = 0 s mm^−2^ acquisition, and one repetition.

Before data pre-processing, the raw acquisitions from all participants were visually inspected to discard any brain lesions, critical head motion, or artifacts that could compromise the data.

### MRI Data Pre-processing

#### Resting-State fMRI

Preprocessing of fMRI data was performed using custom scripts using FMRIB Software Library (FSL v6.01) tools (37). In order to exclude possible magnetic field inhomogeneities at the beginning of the acquisition, the first step of preprocessing was the removal of the first 5 volumes (10 s). Then, because fMRI is measured using 2D imaging, there are temporal offsets between volumes that needed to be corrected. The slice timing correction was done with a temporal data interpolation that used the first acquired volume as reference. The next step was correcting the head motion, by using a function from FSL that searches motion parameters and uses them to correct movement in the time-series. Motion correction is an important step in fMRI preprocessing as the slightest movement can induce motion related artifacts. To further reduce contamination on functional connectivity due to motion, motion scrubbing was also performed to identify and exclude time-points where head motion could be critical. A non-linear spatial normalization to Montreal Neurological Institute (MNI) standard space was performed to register the set of brains to a common space/template. The procedure involved: (i) skull stripping of the mean image of the functional acquisition; (ii) rigid-body registration of the mean functional image to the skull stripped structural scan; (iii) affine registration of the structural scan to the MNI T1 template; (iv) non-linear registration of the structural scan to the MNI T1 template using the affine transformation estimated previously as the initial alignment; (v) non-linear transformation of the functional acquisition to MNI standard space through the sequential application of the rigid-body transformation, followed by non-linear warp and resampling to 2 × 2 × 2 mm^3^ voxel size. A linear regression using the mean of white matter and cerebrospinal fluid (CSF) signals as well as motion outliers was performed, and the residuals of the regression were smoothed using a Gaussian kernel smoother with a full width half maximum of 6 mm (σ = 2.55 mm), band-pass temporal filtered (0.01–0.08 Hz) and used for the subsequent analysis.

#### Resting-State Networks

Probabilistic independent component analysis (PICA) was performed with MELODIC (Multivariate Exploratory Linear Optimized Decomposition into Independent Components), distributed with FSL v6.01. PICA is a data driven analysis that isolates components or non-overlapping spatial maps corresponding to regions evidencing coherent time-courses (38, 39). The software estimates group-wise spatial maps that correspond mainly to Resting State Networks (RSNs). A total number of 30 independent components were used. In order to study subject-specific components, a dual regression analysis was performed as well.

Twelve RSNs (Supplementary Figure 2) of interest were identified by visual inspection in combination with spatial correlations between the obtained components and RSN templates available from a public dataset from the Functional Imaging in Neuropsychiatric Disorders (FIND) Lab at Stanford University (40).

#### Matrices of Functional Connectivity

The matrices of functional connectivity were constructed using the 116 cortical, subcortical, and cerebellar areas of the Anatomical Automatic Labeling (AAL) atlas (28). The mean signal for each brain region was extracted and the Pearson's correlations coefficients between each possible pair of regions were calculated, originating a symmetric adjacency matrix, R. Coefficients in matrix R were then transformed to Z-scores using Fisher's r-to-z transform.

#### DWI Image Pre-processing and Tensor Fitting

Diffusion data were pre-processed using the FMRIB Diffusion Toolbox (FDT) provided with FSL v6.0.3. DWI images were corrected for motion artifacts and eddy current distortions. Then, the affine transformations were used to register each volume and were applied to rotate gradient vectors. Afterwards, the first *b*_0_ volume of each subject was extracted and skull stripped, creating a brain mask applied to the remaining volumes to remove non-brain structures.

Tensor fitting and the scalar maps computation steps were performed with DTIFIT, also included in the FDT toolbox. In this step, a diffusion tensor model is fitted at each voxel and scalar maps of FA and Mean Diffusivity (MD), as well as eigenvector and eigenvalue maps, were generated. Axial Diffusivity (AD) was defined as the principal diffusion eigenvalue, and RD was computed using the mean of the second and third eigenvalues.

#### Tract-Based Spatial Statistics

Voxel-wise analyses of the scalar maps between subjects were performed using TBSS procedures (41), also part of FSL. To remove potential outliers from the tensor fitting, all FA templates were slightly eroded, and the end slices were zeroed. Afterward, all the FA templates were non-linearly registered into a 1-mm × 1-mm × 1-mm standard space. This step was performed by non-linearly registering each subject's FA template to each other to find the “most representative one” (i.e., the one that requires the least warping to align all images), subsequently used as the study-specific target image. Next, the selected target image was affine transformed into the MNI 152 standard space, and each subject's FA template was transformed into this standard space by combining the non-linear transformation to the study-specific target with the affine transformation into the MNI space. Then, the FA templates of all subjects were averaged, and the resulting image skeletonized. After visual inspection of the skeletonized image, we threshold it at 0.35 to remove from the skeleton regions encompassing other tissues, such as gray matter or CSF. Finally, all scalar maps (FA, AD, MD, and RD) were projected into this FA skeleton using the same transformation applied to the FA templates.

### Statistics

#### Psychometric and Demographic Data

Descriptive statistics of psychometric and demographic data were performed using IBM^®^ SPSS^®^ Statistics (version 27; IBM Corp., Armonk, NY, United States). Normality was assessed using the Shapiro-Wilk Test. All demographic and psychometric variables followed a normal distribution except for the BSSI scale where the distribution was tailed toward left-hand. Correlations between non-parametric variables were performed using the Kendall's tau (τ). Results were considered significant for a *p-value* below 0.05.

#### RSN Analysis

RSN FC were correlated with the standardized score (z-score) obtained in the BSSI scale using a regression model with the non-parametric permutation procedure implemented in the *randomise* tool from FSL (42). Threshold-free cluster enhancement (TFCE) was used to detect widespread significant differences and control the family-wise error rate (FWE-R) at α = 0.05. 5,000 permutations were performed for each contrast. Results were corrected for sex, age, and years of education.

#### Network Based Statistics

To assess whether suicidal ideation (BSSI score) was associated with the functional connectome, a regression model using the Network Based Statistics (NBS) was applied (31). The NBS method provides a correction for multiple comparisons equivalent to FWE-R by estimating the probability of identifying in a random permutation of the data, networks larger in extent than the ones identified by the hypothesis tested. The method consists in two parts. In the first part, the statistical hypothesis is tested for each connection, filtered by a user-chosen statistical edge threshold and significant network components are identified. In a significant network component, a node can be reached from any other through significant connections. The size of the component is calculated by the number of significant connections in it and although the user-defined threshold is not a determinant of the significance of the network, larger thresholds reveal more widespread networks and smaller thresholds produce smaller and more focused results. A set of different primary thresholds (0.005, 0.001, 0.0005 and 0.0001) was used, as suggested by the toolbox authors, to access different levels of sensibility. In the second part, random permutations of the data were created, and the same methodology described in the first part is applied to each permutation and the size of the network components found is calculated. The calculated distribution of components size is used to estimate the probability of finding random components with a size greater than the one found in our hypothesis. A total of 5,000 permutations were used, together with a FWE corrected network significance of 0.05. Results were corrected for sex, age, and years of education.

#### Diffusion Data

In a similar way to the analyses performed with RSN FC, non-parametric permutation methods were employed using the *randomise* tool from FSL (42), in order to analyze the skeletonized maps of FA, AD, MD, and RD. Particularly, linear regression models were performed to investigate whether WM microstructure measures were associate with suicidal ideation (z-scores of BSSI score). These models were adjusted for age, gender/sex and years of education.

Widespread significant differences were detected with TFCE, and multiple comparisons were corrected using FWE-R at α = 0.05 and cluster extent threshold of *K* > 50. Clusters showing significant results were identified using the John Hopkins University ICBM-DTI-81 WM Labels Atlas and dilated with the *tbss_fill* tool (included in FSL) for visualization purposes.

## Results

### Psychometric and Demographic Data

Descriptive statistics of psychometric and demographic data are detailed in Table 1. Our cohort was composed of 25 drug-naïve MDD patients (9 males and 16 females) with an age mean of 37 years (ranging from 19 to 59 years of age). In our sample 10 patients presented no current suicidal ideation (BSSI score = 0) and 22 patients presented no previous history of suicide attempt.

**Table 1:** Demographic and psychological characterization of the sample.

	***N***	**Mean**	**Std. error mean**	**95% CI lower**	**95% CI upper**	**Min**	**Max**
Age (years)	25	37.44	2.47	32.35	42.53	19	59
Sex (Male/Female)	9/16						
Education (years)	25	11.64	0.99	9.6	13.68	4	20
HAM-D	25	20.72	1.45	17.72	23.72	9	33
HAM-A	25	22.96	1.77	19.3	26.62	8	40
PSS-10	25	27.36	1.07	25.15	29.57	17	36
BSSI	25	5.12	1.46	2.1	8.14	0	23
Previous SA	3						

*HAM-D, Hamilton Depression Rating Scale; HAM-A, Hamilton Anxiety Rating Scale; PSS-10, Perceived Stress Scale 10 items; BSSI, Beck Suicidal Ideation Scale; SA, suicide attempts; CI, confidence interval; Min, minimum; Max, maximum*.

In our cohort, suicidal ideation (BSSI) was not found to be significantly correlated with depression (HAM-D, τ = 0.155, *p* = 0.375), anxiety (HAM-A, τ = 0.069, *p* = 0.659) or perceived stress (PSS-10, τ = 0.083, *p* = 0.602) as well as demographic variables such as years of education (τ = 0.165, *p* = 0.292) or age (τ = 0.101, *p* = 0.511).

### RS-FC Correlates of Suicidal Ideation in Drug-Naïve MDD

#### Resting State Networks

Thirty independent components were obtained using the MELODIC approach. From these components, 12 were consistent with the typical and identifiable RSN (Supplementary Figure 2) (43).

**Figure 1:**
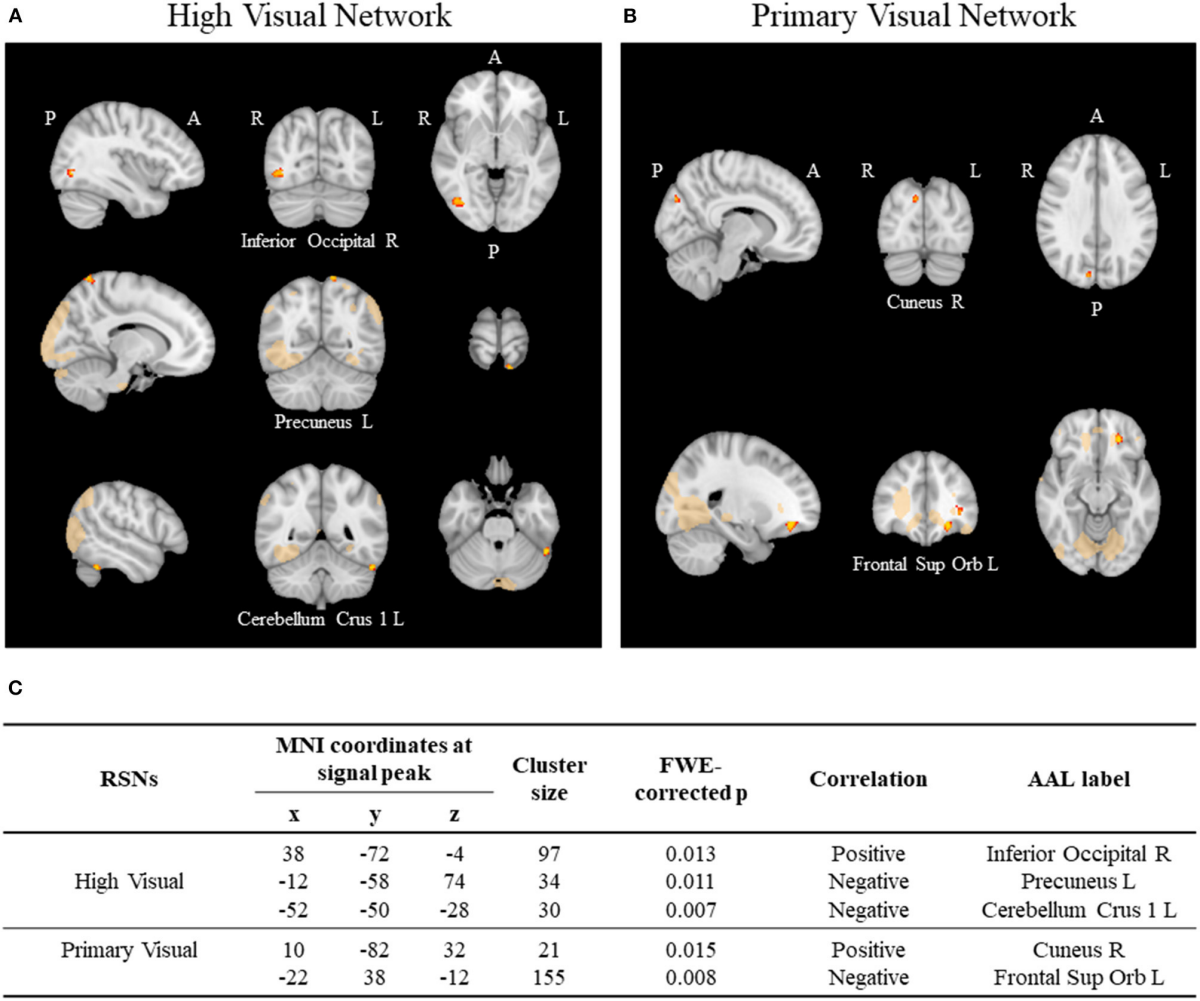
Resting state networks (RSNs) using ICA. **(A)** Significant correlations between suicidal ideation and functional connectivity with the high visual network and the **(B)** primary visual network. Significance threshold was set to *p* < 0.05 (FWE-R correction for multiple comparisons). **(C)** Table illustrating the clusters positively and negatively associated with suicidal ideation score in each network.

Our results revealed positive and negative correlations between suicidal ideation and functional connectivity to the primary and high visual networks (Figure 1). Statistical analysis revealed that in our cohort of drug naïve MDD patients, the FC between the high visual network and the right inferior occipital gyrus was positively correlated with suicidal ideation (FWE-corrected *p* value = 0.013, Figures 1A,C). In the other hand, the FC between the same network and the left precuneus and cerebellum were negatively correlated with suicidal ideation (FWE-corrected *p*-value = 0.011, FWE-corrected *p*-value = 0.007, respectively, Figures 1A,C). As for the primary visual network, the connectivity to the right cuneus had a positive association with suicidal ideation, inversely the connectivity to the frontal superior orbital gyrus was negatively correlated (FWE-corrected *p*-value = 0.015, FWE-corrected *p*-value = 0.007, respectively, Figures 1B,C). Figure 1C presents details on statistical analysis obtained for each cluster (including MNI coordinates, FWE-corrected peak *p*-values and cluster sizes).

#### Network-Based Statistics

**Figure 2:**
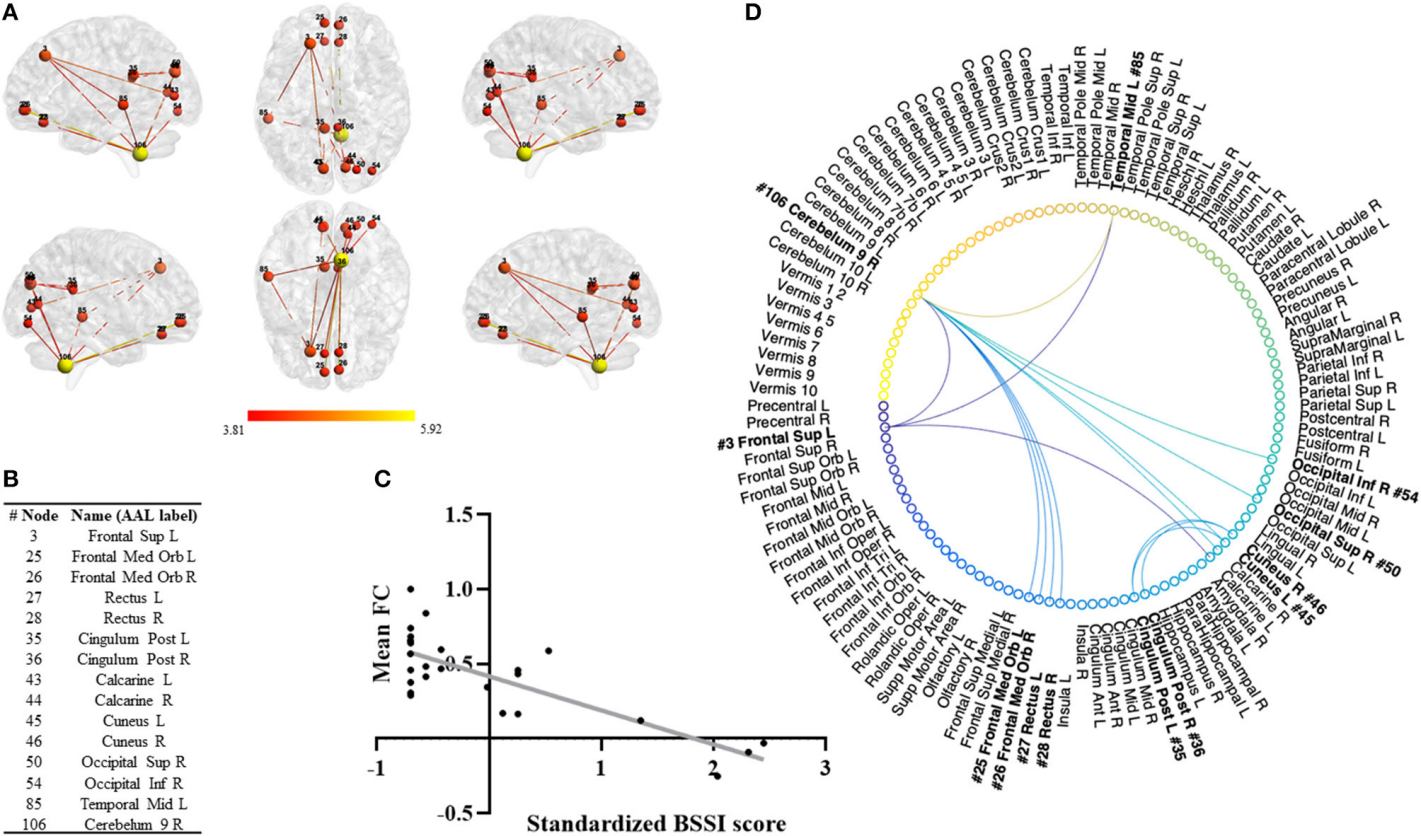
Network Based Statistics (NBS). **(A)** Significant negative correlation between suicidal ideation and a functional connectivity network (adjusted for age, sex, and years of education) using the NBS method. Network presented for the significant threshold *p* = 0.001, t_(threshold)_ =3.78, df = 23, *p*-value (network) = 0.025, 15 nodes and 15 edges. **(B)** Nodes comprising the network and the correspondent AAL label. **(C)** Scatterplot illustrating the negative correlation between FC and suicidal ideation for each patient. The x-axis represents average values of FC and y-axis the standardized suicide ideation severity score on BSSI. The gray line represents the overall regression line. **(D)** Circular graphical representation of the nodes and edges of the network.

From the connectivity analysis performed using the NBS method, a single network was found to be significantly correlated with suicidal ideation (BSSI). In our cohort of MDD patients, a higher FC in this network was related to lower scores in the BSSI scale. The NBS analysis was performed using thresholds between 0.005 and 0.0001 (Supplementary Tables 1, 2). In Figure 2, we present the results found for the significant threshold *p*-value = 0.001 t_(threshold)_ = 3.78, df = 23, *p*-value (network) = 0.025, 15 nodes and 15 edges. From the set of tested thresholds this was the network that showed a higher mean FC for the lowest *p*-value.

Of this network we emphasize the connections between the cerebellum (node #106) and the nodes in the left superior frontal gyrus (node #3), the frontal middle orbital cortex (nodes #25 and #26), the rectus (nodes #27 and #28) and the right inferior and superior occipital gyrus (nodes #54 and #50). This network also presented relevant connections between the left superior frontal gyrus (node #3) and the left middle temporal gyrus (node #85) and the cuneus (nodes #45 and #46) and the posterior cingulate gyrus (nodes #35 and #36).

#### WM Microstructure Correlates of Suicidal Ideation in Drug-Naïve MDD

**Figure 3:**
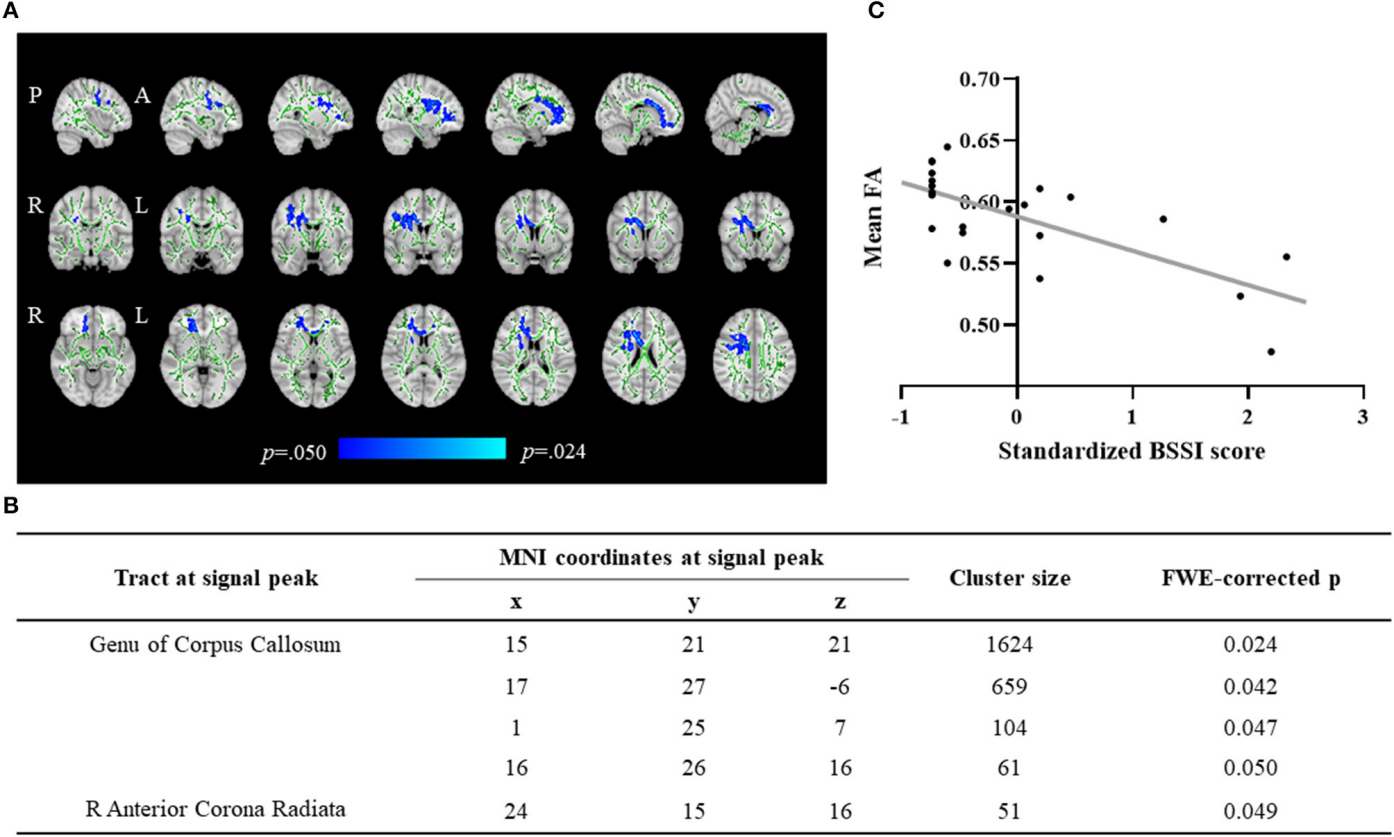
Tract-based spatial statistics (TBSS). **(A)** Significant negative correlation between suicidal ideation and FA maps in drug-naïve patients with Major Depression (adjusted for age, sex, and years of education). Blue/light-blue voxels indicate a significant decrease in FA. Significance threshold was set to *p* < 0.05 (FWE-R correction for multiple comparisons). The WM skeleton (represented in green) is superimposed on a T1-weighted MNI template. **(B)** Table illustrating the clusters negatively associated with suicidal ideation score. **(C)** Scatterplot illustrating the negative correlation between FA of the significant clusters and suicidal ideation score. The x-axis represents average values of FA and y-axis the standardized suicide ideation severity score on BSSI. The gray line represents the overall regression line.

The statistical analysis of the skeletonized maps revealed a statistically significant negative correlation between suicidal ideation scores and FA maps (Figure 3A). Patients with higher suicidal ideation scores showed a significant decrease in FA maps in clusters including the genu of the Corpus Callosum and the right anterior Corona Radiata (Figure 3B). Figure 3C presents details on statistical analysis obtained for each cluster (including MNI coordinates, FWE-corrected peak *p*-values and cluster sizes). No significant correlations were found between suicidal ideation score and AD, MD, and RD maps.

## Discussion

The present study aimed to investigate the neuronal correlates of suicidal ideation in drug-naïve depressed patients using a multimodal MRI approach, specifically resting-state fMRI, and DTI. In our sample of MDD patients, three (12%) reported previous history of suicide attempt, whereas in previous studies including drug-naïve depressed patients 16%-20% reported suicide attempts in the past (44–46).

Our results showed that, in our cohort of MDD patients, suicidal ideation was negatively correlated with rsFC in the visual networks and FA in the genu of corpus callosum and the right anterior CR. Moreover, we identified a specific rsFC network associated with suicidal ideation that included connections between regions in the superior and orbitofrontal cortex, the cerebellum, as well as temporal and occipital regions. Moreover, patients' scores on HAM-D were not correlated with BSSI scores, suggesting that in our cohort of patients with depression, higher suicidal ideation is not associated with higher depression severity.

Regarding the functional correlates of suicidal ideation in MDD, the results in our cohort showed that suicidal ideation was correlated with functional connectivity with the visual RSN, both high and primary visual networks. These results seem to indicate a dysregulation in rsFC involving brain regions such as the right inferior occipital gyrus (positive correlation), the left precuneus and cerebellum (negative correlation) and their interaction with the regions that compose the high visual network. In accordance, the connectivity between primary visual network and the right cuneus was positively correlated with suicidal ideation. Finally, the connectivity between left frontal superior orbital gyrus and the primary visual network was negatively correlated with the suicidal ideation in MDD. These results in the RSN seem to be in line with the ones obtained using a different approach to FC. In fact, the NBS method revealed a network of FC that was also shown to be negatively correlated with suicidal ideation in our cohort of MDD patients before the initiation of an antidepressant. The regions of this network overlap, to some extent, with the ones previously found using the typical RSNs, reinforcing the idea of their involvement in the suicidal ideation processes. The areas that compose this network range from regions involved in higher order cognitive functions (such as the medial frontal orbital gyrus, left frontal superior gyrus, posterior cingulate and rectus) to visual processing functions (such as the calcarine, superior and inferior left occipital gyrus and cuneus) and semantic and conceptual processing (left middle temporal gyrus) and motor control (cerebellum).

In a previous study using NBS, Chen et al. reported a specific network of FC that distinguished MDD patients with and without suicidal ideation (23). The reported network was composed by areas that overlap partially with the network found in our cohort, specifically the orbital frontal gyrus, the posterior cingulate cortex, the rectus, and the occipital gyrus. Moreover, another study by Kim et al., also using NBS (24), reported a network of FC that distinguished MDD patients with and without suicidal ideation. That network also presents some regions that overlap with our observations, specifically the left middle temporal gyrus, the middle frontal orbitofrontal gyrus and the superior frontal gyrus. Thus, together with previous evidence, our results seem to indicate a possible involvement of a specific network of rsFC in the suicidal ideation processes.

Differently, our results showed a possible involvement of the cerebellum in the network of functional connectivity associated with suicidal ideation. The association between the cerebellum and suicidal ideation has been previously hypothesized, however its involvement in the process is still unclear. Our findings seem to be in line with previous studies that revealed that the cerebellum plays a role in higher order cognitive processes but also mood regulation in disorders such as major depression and bipolar disorder (BD) (47, 48) with reported reduced cerebellar volumes in BD patients with previous suicide attempts (49). Also in line with our results, Shaffer et al. suggested a role of the cerebellum in the neuronal circuits involved in suicidal behavior (50).

Regarding the white matter microstructure, our findings revealed that increased suicidal ideation was associated with decreased FA in the genu of corpus callosum and the anterior CR, mainly in the right hemisphere. These findings suggest that alterations in the white matter microstructure are associated with higher suicidal ideation in MDD. Taylor et al. (29) also suggested alterations in the white matter microstructure in depressed patients with suicidal ideation, although they reported widespread alterations in CgH, superior and posterior CR, and ATR. The incongruent findings between the studies might be explained by the characteristics of the sample (first depressive episode vs. recurrent depressive episodes), previous exposure to antidepressants (drug-naïve vs. drug washout for at least 1 month), or even the characteristics of the diffusion acquisition (30 directions vs. 20 directions). Moreover, in line with our results, Chen and colleagues (30) reported decreased generalized fractional anisotropy and normalized quantitative anisotropy values in corpus callosum and anterior cingulate in depressed patients with suicidal ideation, suggesting focal alterations in the white matter integrity associated with suicidal ideation.

The genu of the corpus callosum and the anterior CR were the 2 major white matter structures displaying a significant association with suicidal ideation. The genu of the corpus callosum comprises fibers from orbital, medial, and dorsolateral frontal cortex, whereas the anterior CR is formed by thalamic and motor projections from the internal capsule to the cortex (51). Previous studies reported a decreased FA in the genu of the corpus callosum associated with an increased number of suicide attempts in depressed and bipolar patients (52), as well as reduced number of projecting fibers from the internal capsule to orbitofrontal cortex and thalamus in depressed suicide attempters when compared with depressed controls (53). Taken together with our findings, these observations suggest that alterations in the white matter microstructure and connections of these bundles might contribute to an increased suicide risk, given its association with both suicidal ideation and behaviors. Also supporting this hypothesis, Myung and collaborators (28) described that depressed patients with suicidal ideation present a reduced structural connectivity in a specific network, including the orbitofrontal cortex and thalamus.

The present work has expanded our knowledge into the possible neuronal correlates of suicidal ideation in MDD patients before antidepressant medication is initiated. Besides the alterations in the white-matter integrity (in the genu of corpus callosum and the right anterior CR), our results also revealed abnormal functional connectivity in the visual RSN but also in a specific network composed by frontal, cerebellar, occipital, and parietal regions. Importantly, in our cohort of MDD patients, suicidal ideation was not found to be associated with worse depression severity and thus suggesting that these results were specific to suicidal ideation. It is often difficult to separate the suicidal ideation process from factors such as disease severity, duration of illness and medication status as these variables are often clinically linked and not independent.

The present study presents some important limitations, first being the limited sample size and the strength of the MRI magnetic field being 1.5 Tesla that limited the quality of the signal and the number of directions considered in the DTI acquisitions, as well as the absence of a control group. Due to these limitations, caution should be applied when interpreting these results as more studies should be done in the sense of replication of the patterns presented in this paper. The present study shared some light into the brain alterations associated with suicidal ideation in MDD patients. Such knowledge could be at use in the guidance of better treatments that could target deregulations linked to suicidal ideation in MDD patients.

## Data Availability Statement

The raw data supporting the conclusions of this article will be made available by the authors, without undue reservation.

## Ethics Statement

The studies involving human participants were reviewed and approved by Ethics Committee of Hospital of Braga and Ethics Committee of University of Minho. The patients/participants provided their written informed consent to participate in this study.

## Author Contributions

JR, RV, AC, RM, PM, and SF planned and performed the MRI scanning seasons. JR, RV, and AC did the MRI processing and performed the statistical analysis of the data. RV and CP-N maintained the database and organized the evaluation sessions and collected the psychometric and demographic data. JB recruited the participants. JR and RV wrote the first draft of the manuscript. JB, ND, and NS conceived and designed the study. All authors participated in the data analysis and discussions. All authors revised the manuscript. All authors contributed to the article and approved the submitted version.

## Funding

This work was funded by the Portuguese Foundation for Science and Technology (FCT) under scope of project PTDC/DTP-PIC/6936/2014. This work has been funded by National Funds, through the Foundation for Science and Technology (FCT)—Project UIDB/50026/2020 and UIDP/50026/2020 and by the project NORTE-01-0145-FEDER-000039, supported by Norte Portugal Regional Operational Program (NORTE 2020), under the PORTUGAL 2020 Partnership Agreement, through the European Regional Development Fund (ERDF). JR was supported by the FCT Fellowship Grant with the reference PDE/BDE/113602/2015 from the PhD-iHES program. RV was supported by the FCT Fellowship Grant with the reference PD/BDE/150619/2020 from the PhD-iHES program and UMINHO/BI/340/2018. CP-N was supported by the Portuguese Science Foundation (FCT) Doctoral Scholarship PD/BD/106050/2015 *via* Inter-University PhD Program in Aging and Chronic Diseases. PM was supported by the FCT Fellowship Grant with the reference PDE/BDE/113601/2015 from the PhD-iHES program. RM was supported by the FCT Fellowship Grant with the reference PDE/BDE/113604/2015 from the PhD-iHES program. SF was supported by the FCT Fellowship Grant with the reference PD/BDE/127839/2016 from the PhD-iHES program.

## Conflict of Interest

The authors declare that the research was conducted in the absence of any commercial or financial relationships that could be construed as a potential conflict of interest.

## Publisher's Note

All claims expressed in this article are solely those of the authors and do not necessarily represent those of their affiliated organizations, or those of the publisher, the editors and the reviewers. Any product that may be evaluated in this article, or claim that may be made by its manufacturer, is not guaranteed or endorsed by the publisher.

## References

[1] Abbafati C Abbas K Abbasi-Kangevari M Abd-Allah F Abdelalim A Abdollahi MC. Global burden of 369 diseases and injuries in 204 countries and territories, 1990–2019: a systematic analysis for the Global Burden of Disease Study 2019. Lancet. (2020) 396:1204–22. 10.1016/S0140-6736(20)30925-9 33069326 PMC7567026

[2] World Health Organization. Depression and Other Common Mental Disorders: Global Health Estimates. Geneva: World Health Organization (2017).

[3] Luo M Guo L Yu M Wang H. The psychological and mental impact of coronavirus disease 2019 (COVID-19) on medical staff and general public—a systematic review and meta-analysis. Psychiatry Res. (2020) 291:113190. 10.1016/j.psychres.2020.113190 32563745 PMC7276119

[4] Bueno-Notivol J Gracia-García P Olaya B Lasheras I López-Antón R Santabárbara J. Prevalence of depression during the COVID-19 outbreak: A meta-analysis of community-based studies. Int J Clin Health Psychol. (2021) 21:100196. 10.1016/j.ijchp.2020.07.007 32904715 PMC7458054

[5] American Psychiatric Association. Diagnostic and Statistical Manual of Mental Disorders (5th ed). Washington, DC: APA (2013).

[6] Ferrari A Charlson F Norman R Patten S Freedman G Murray C. Burden of depressive disorders by country, sex, age, and year: findings from the Global Burden of Disease Study 2010. PLoS Med. (2013) 10:e1001547. 10.1371/journal.pmed.1001547 24223526 PMC3818162

[7] Nock MK Hwang I Sampson N Kessler RC Angermeyer M Beautrais A. Cross-national analysis of the associations among mental disorders and suicidal behavior: findings from the WHO World Mental Health Surveys. PLoS Med. 6:e1000123. 10.1371/journal.pmed.1000123 19668361 PMC2717212

[8] Lépine JP Briley M. The increasing burden of depression. Neuropsychiatr Dis Treat 7. (2011) 3–7. 10.2147/NDT.S19617 21750622 PMC3131101

[9] Ösby U Brandt L Correia N Ekbom A Sparén P. Excess mortality in bipolar and unipolar disorder in Sweden. Arch Gen Psychiatry. (2001) 58:844–50. 10.1001/archpsyc.58.9.844 11545667

[10] Kessler RC. The costs of depression. Psychiatr Clin North Am. (2012) 35:1–14. 10.1016/j.psc.2011.11.005 22370487 PMC3292769

[11] World Health Organization (WHO). Suicide Worldwide in 2019: Global Health Estimates. Geneva: WHO (2021).

[12] Crosby A Han B Ortega L Parks S Gfroerer J CDC. Suicidal thoughts and behaviors among adults aged ≥18 years—United States, 2008–2009. MMWR Surveill Summ. (2011) 60:1–22. 10.15585/mmwr.ss7101a1 22012169

[13] Wasserman D. The suicidal process. In: Suicide: An unnecessary death. Oxford: Oxford University Press (2016). p. 27–38.

[14] Orsolini L Latini R Pompili M Serafini G Volpe U Vellante F. Understanding the complex of suicide in depression: From research to clinics. Psychiatry Investig. (2020) 17:207–21. 10.30773/pi.2019.0171 32209966 PMC7113180

[15] Franklin J Ribeiro J Fox K Bentley K Kleiman E Huang X. Risk factors for suicidal thoughts and behaviors: a meta-analysis of 50 years of research. Psychol Bull. (2017) 143:187–232. 10.1037/bul0000084 27841450

[16] Lopez-Castroman J Jaussent I Gorwood P Courtet P. Suicidal depressed patients respond less well to antidepressants in the short term. Depress Anxiety. (2016) 33:483–94. 10.1002/da.22473 26882201

[17] Marzuk PM Hartwell N Leon AC Portera L. Executive functioning in depressed patients with suicidal ideation. Acta Psychiatr Scand. (2005) 112:294–301. 10.1111/j.1600-0447.2005.00585.x 16156837

[18] Pu S Setoyama S Noda T. Association between cognitive deficits and suicidal ideation in patients with major depressive disorder. Sci Rep. (2017) 7:1–6. 10.1038/s41598-017-12142-8 28912439 PMC5599636

[19] Lan X Zhou Y Zheng W Zhan Y Liu W Wang C. Association between cognition and suicidal ideation in patients with major depressive disorder: a longitudinal study. J Affect Disord. (2020) 272:146–51. 10.1016/j.jad.2020.03.141 32379606

[20] Nock M Borges G Bromet E Alonso J Angermeyer M Beautrais A. Cross-national prevalence and risk factors for suicidal ideation, plans and attempts. Br J Psychiatry 192. (2008) 98–105. 10.1192/bjp.bp.107.040113 18245022 PMC2259024

[21] Kleber H Weiss R Anton R George T Greenfield S Kosten T. Treatment of patients with substance use disorders, second edition. Am J Psychiatry. (2007). 164 (4 Sup):5–123. 17569411

[22] Schmaal L Pozzi EC Ho T van Velzen L Veer I Opel N. ENIGMA MDD: seven years of global neuroimaging studies of major depression through worldwide data sharing. Transl Psychiatry. (2020) 10:1–19. 10.1038/s41398-020-0842-6 32472038 PMC7260219

[23] Chen V Chou Y Tsai Y Huang Y McIntyre R Weng J. Resting-state functional connectivity and brain network abnormalities in depressive patients with suicidal ideation. Brain Topogr. (2021) 34:234–44. 10.1007/s10548-020-00817-x 33420533

[24] Kim K Kim S Myung W Han C Fava M Mischoulon D. Reduced orbitofrontal-thalamic functional connectivity related to suicidal ideation in patients with major depressive disorder. Sci Rep. (2017) 7:1–11. 10.1038/s41598-017-15926-0 29150619 PMC5693996

[25] Wei S Chang M Zhang R Jiang X Wang F Tang Y. Amygdala functional connectivity in female patients with major depressive disorder with and without suicidal ideation. Ann Gen Psychiatry. (2018) 17:1–7. 10.1186/s12991-018-0208-0 30214465 PMC6134510

[26] Qiao D Zhang A Sun N Yang C Li J Zhao T. Altered static and dynamic functional connectivity of habenula associated with suicidal ideation in first-episode, drug-naïve patients with major depressive disorder. Front Psychiatry. (2020). 1439:608197. 10.3389/fpsyt.2020.608197 33391057 PMC7772142

[27] Du L Zeng J Liu H Tang D Meng H Li Y. Fronto-limbic disconnection in depressed patients with suicidal ideation: a resting-state functional connectivity study. J Affect Disord. (2017) 215:213–7. 10.1016/j.jad.2017.02.027 28340447

[28] Myung W Han C Fava M Mischoulon D Papakostas G Heo J. Reduced frontal-subcortical white matter connectivity in association with suicidal ideation in major depressive disorder. Transl Psychiatry. (2016) 6:e835–e835. 10.1038/tp.2016.110 27271861 PMC4931608

[29] Taylor W Boyd B McQuoid D Kudra K Saleh A MacFall J. Widespread white matter but focal gray matter alterations in depressed individuals with thoughts of death. Prog Neuro-Psychopharmacol Biol Psychiatry. (2015) 62:22–8. 10.1016/j.pnpbp.2015.05.001 25963377 PMC4458419

[30] Chen V Kao C Tsai Y Cheok M McIntyre R Weng J. Assessment of disrupted brain structural connectome in depressive patients with suicidal ideation using generalized Q-sampling MRI. Front Hum Neurosci. (2021) 15:711731. 10.3389/fnhum.2021.711731 34512298 PMC8430248

[31] First MB Williams JBW Karg RS Spitzer RL Gibbon M. Structured Clinical Interview for DSM-IV Axis I Disorders (SCID-I). New York: Elsevier (2008).

[32] Folstein MF Folstein SE McHugh PR. ‘Mini-mental state’. A practical method for grading the cognitive state of patients for the clinician. J Psychiatr Res. (1975) 12:189–98. 10.1016/0022-3956(75)90026-6 1202204

[33] Hamilton M. A rating scale for depression. J Neurol Neurosurg Psychiatry. (1960) 23:56–62. 14399272 10.1136/jnnp.23.1.56 PMC495331

[34] Hamilton M. (1959). The assessment of anxiety states by rating. Br J Med Psychol 32:50–5. 10.1111/j.2044-8341.1959.tb00467.x 13638508

[35] Cohen S. Perceived stress in a probability sample of the United States. In: Spacapan S, Oskamp S, editors. The Social Psychology of Health. Sage Publications, Inc. (1988). p. 31–67.

[36] Beck AT Steer RA. Manual for the Beck Scale for Suicide Ideation. New York, NY: Psychological Corporation (1991).

[37] Smith S Jenkinson M Woolrich M Beckmann C Behrens T Johansen-Berg H. Advances in functional and structural MR image analysis and implementation as FSL. Neuroimage. (2004) 23:S208–19. 10.1016/j.neuroimage.2004.07.051 15501092

[38] Soares JM Magalhães R Moreira P Sousa A Ganz E Sampaio A. A Hitchhiker's guide to functional magnetic resonance imaging. Front Neurosci. (2016) 10:515. 10.3389/fnins.2016.00515 27891073 PMC5102908

[39] Beckmann CF Smith SM. Probabilistic independent component analysis for functional magnetic resonance imaging. IEEE Trans Med Imaging. (2004) 23:137–52. 10.1109/TMI.2003.822821 14964560

[40] Shirer WR Ryali S Rykhlevskaia E Menon V Greicius MD. Decoding subject-driven cognitive states with whole-brain connectivity patterns. Cereb Cortex. (2012) 22:158–65. 10.1093/cercor/bhr099 21616982 PMC3236795

[41] Smith S Jenkinson M Johansen-Berg H Rueckert D Nichols T Mackay C. Tract-based spatial statistics: Voxelwise analysis of multi-subject diffusion data. Neuroimage. (2006) 31:1487–505. 10.1016/j.neuroimage.2006.02.024 16624579

[42] Winkler AM Ridgway GR Webster MA Smith SM Nichols TE. Permutation inference for the general linear model. Neuroimage. (2014) 92:381–97. 10.1016/j.neuroimage.2014.01.060 24530839 PMC4010955

[43] Damoiseaux J Rombouts S Barkhof F Scheltens P Stam C Smith S. Consistent resting-state networks across healthy subjects. Proc Natl Acad Sci. (2006) 103:13848–53. 10.1073/pnas.0601417103 16945915 PMC1564249

[44] Yang W Zhang G Jia Q Qian Z Yin G Zhu X. Prevalence and clinical profiles of comorbid anxiety in first episode and drug naïve patients with major depressive disorder. J Affect Disord. (2019) 257:200–6. 10.1016/j.jad.2019.06.052 31301624

[45] Zhao K Zhou S Shi X Chen J Zhang Y Fan K. Potential metabolic monitoring indicators of suicide attempts in first episode and drug naive young patients with major depressive disorder: a cross-sectional study. BMC Psychiatry. (2020) 20:387. 32723375 10.1186/s12888-020-02791-x PMC7389868

[46] Zhou Y Li Z Wang Y Huang H Chen W Dong L. Prevalence and clinical correlates of psychotic depression in first-episode and drug-naïve outpatients with major depressive disorder in a Chinese Han population. J Affect Disord. (2020) 263:500–6. 10.1016/j.jad.2019.10.051 31759662

[47] Schmahmann JD. The role of the cerebellum in cognition and emotion: Personal reflections since 1982 on the dysmetria of thought hypothesis, and its historical evolution from theory to therapy. Neuropsychol Rev. (2010) 20:236–60. 10.1007/s11065-010-9142-x 20821056

[48] Stoodley CJ Schmahmann JD. Evidence for topographic organization in the cerebellum of motor control versus cognitive and affective processing. Cortex. (2010) 46:831–44. 10.1016/j.cortex.2009.11.008 20152963 PMC2873095

[49] Johnson SL Carver CS Tharp JA. Suicidality in bipolar disorder: the role of emotion-triggered impulsivity. Suicide Life-Threatening Behav. (2017) 47:177–92. 10.1111/sltb.12274 27406282 PMC5788807

[50] Shaffer J Willour V Fiedorowicz J Christensen G Long J Johnson C. Distinct patterns of altered quantitative T1ρ and functional BOLD response associated with history of suicide attempts in bipolar disorder. Brain Imaging Behav. (2021) 2021;1–14. 10.1007/s11682-021-00552-2 34601647 PMC8975910

[51] Catani M Howard RJ Pajevic S Jones DK. Virtual *in vivo* interactive dissection of white matter fasciculi in the human brain. Neuroimage. (2002) 17:77–94. 10.1006/nimg.2002.1136 12482069

[52] Cyprien F de Champfleur N Deverdun J Olié E Le Bars E Bonafé A. Corpus callosum integrity is affected by mood disorders and also by the suicide attempt history: a diffusion tensor imaging study. J Affect Disord. (2016) 206:115–24. 10.1016/j.jad.2016.07.026 27472413

[53] Jia Z Wang Y Huang X Kuang W Wu Q Lui S. Impaired frontothalamic circuitry in suicidal patients with depression revealed by diffusion tensor imaging at 3.0 T. J Psychiatry Neurosci. (2014) 39:170–77. 10.1503/jpn.130023 24119793 PMC3997602

