# Supplementary material for: Suicidal Ideation Is Associated With Reduced Functional Connectivity and White Matter Integrity in Drug-Naïve Patients With Major Depression
Source: Front Psychiatry. 2022 Mar 21;13:838111. doi: 10.3389/fpsyt.2022.838111 (PMC8978893; doi:10.3389/fpsyt.2022.838111)
Supplement: Supplementary file 1 [file Data_Sheet_1.docx]

**Supplementary Figure 1.** Detailed information on subject recruitment and exclusion.

30 patients underwent MRI acquisition

32 patients enrolled in the study

Functional MRI data available on **25 patients**;

Diffusion data available on **23 patients.**

Did not initiate MRI due to claustrophobia (n=2).

Found brain lesions or artifacts in the scans (n=2);

Scans were lost (n=1);

No diffusion acquisition (MRI acquisition ended earlier) (n=2).

**Supplementary Figure 2. Resting State Networks.** Resting state networks identified through MELODIC. Group MELODIC maps (LECN and RECN left and right executive networks, dorsal and ventral DMN, posterior and anterior salience networks, primary and high visual networks, sensorimotor network, visuospatial network, precuneus networks and basal ganglia network.


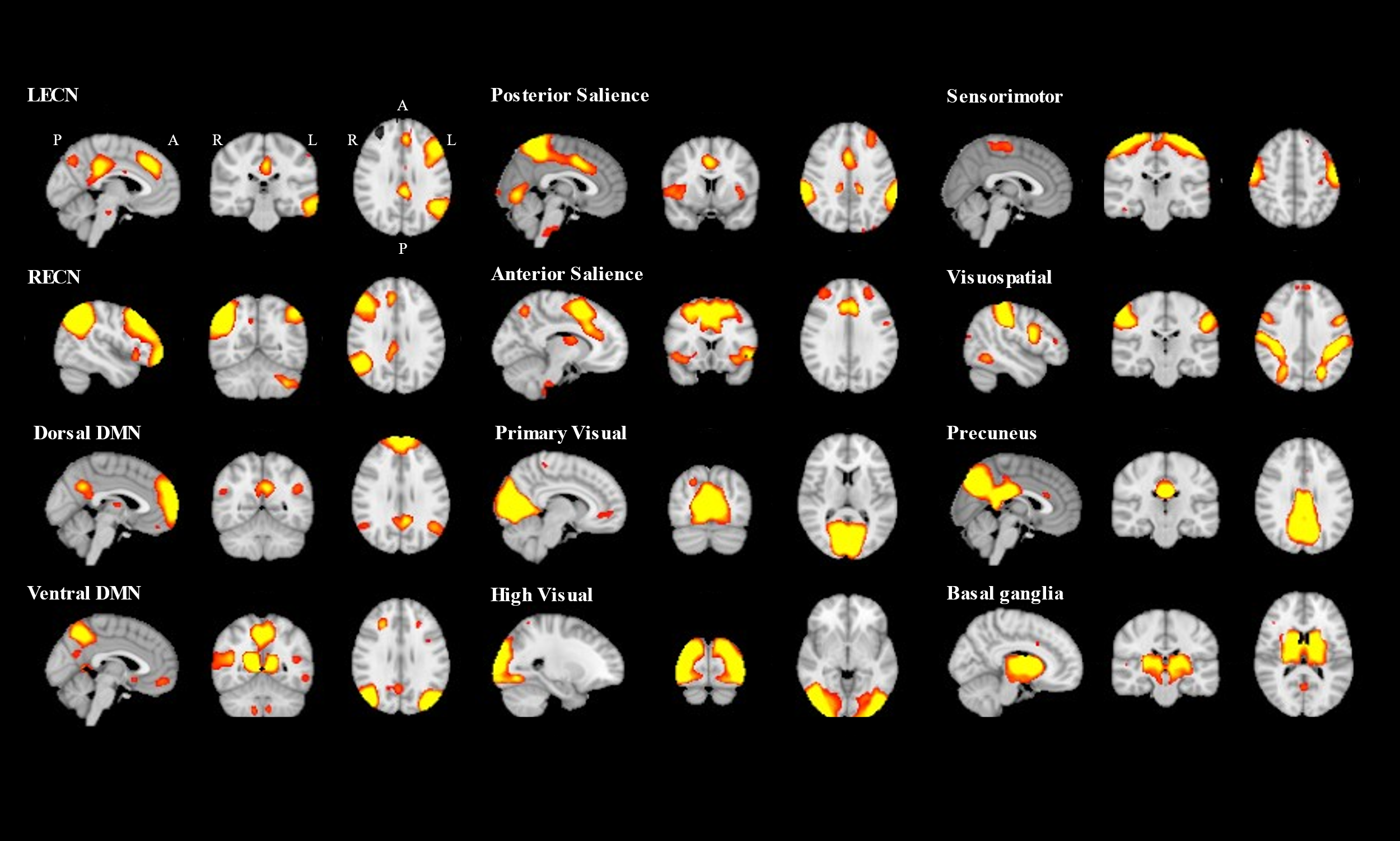


**Supplementary Table 1:** Results of the functional connectivity analysis using NBS. For each threshold used in the NBS analysis, we report the p-value of the significant network, the number of nodes and edges in the network as well as the mean connectivity for all patients.

| **Threshold (t, p)** | **p (network)** | **N. Nodes** | **N. Edges** | **Mean FC** |
| --- | --- | --- | --- | --- |
| 3.10, 0.005 | 0.046 | 44 | 71 | 0.31 |
| 3.78, 0.001 | 0.025 | 15 | 15 | 0.42 |
| 4.05, 0.0005 | 0.029 | 7 | 6 | 0.39 |
| 4.69, 0.0001 | 0.037 | 3 | 2 | 0.41 |

**Supplementary table 2:** Nodes of the networks with connecting edges that have a significant correlation with BSI. Results are represented for the 4 tested thresholds using the AAL atlas and are, name and number of each node as well as the sum t-statistic value (t) over its significant connections.

|  |  | **p-value** | | | |
| --- | --- | --- | --- | --- | --- |
| **# Node** | **Name (AAL label)** | **0.005** | **0.001** | **0.0005** | **0.0001** |
| **1** | Precentral L | 0 | 0 |  | 0 |
| **2** | Precentral R | 0 | 0 | 0 | 0 |
| **3** | Frontal Sup L | 16.09 | 12.501 | 0 | 0 |
| **4** | Frontal Sup R | 0 | 0 | 0 | 0 |
| **5** | Frontal Sup Orb L | 0 | 0 | 0 | 0 |
| **6** | Frontal Sup Orb R | 3.799 | 0 | 0 | 0 |
| **7** | Frontal Mid L | 0 | 0 | 0 | 0 |
| **8** | Frontal Mid R | 0 | 0 | 0 | 0 |
| **9** | Frontal Mid Orb L | 0 | 0 | 0 | 0 |
| **10** | Frontal Mid Orb R | 0 | 0 | 0 | 0 |
| **11** | Frontal Inf Oper L | 0 | 0 | 0 | 0 |
| **12** | Frontal Inf Oper R | 0 | 0 | 0 | 0 |
| **13** | Frontal Inf Tri L | 0 | 0 | 0 | 0 |
| **14** | Frontal Inf Tri R | 0 | 0 | 0 | 0 |
| **15** | Frontal Inf Orb L | 0 | 0 | 0 | 0 |
| **16** | Frontal Inf Orb R | 3.5361 | 0 | 0 | 0 |
| **17** | Rolandic Oper L | 0 | 0 | 0 | 0 |
| **18** | Rolandic Oper R | 0 | 0 | 0 | 0 |
| **19** | Supp Motor Area L | 0 | 0 | 0 | 0 |
| **20** | Supp Motor Area R | 0 | 0 | 0 | 0 |
| **21** | Olfactory L | 0 | 0 | 0 | 0 |
| **22** | Olfactory R | 10.282 | 0 | 0 | 0 |
| **23** | Frontal Sup Medial L | 3.1574 | 0 | 0 | 0 |
| **24** | Frontal Sup Medial R | 3.4138 | 0 | 0 | 0 |
| **25** | Frontal Med Orb L | 19.11 | 5.5733 | 5.5733 | 5.5733 |
| **26** | Frontal Med Orb R | 16.168 | 5.9173 | 5.9173 | 5.9173 |
| **27** | Rectus L | 10.485 | 3.9627 | 0 | 0 |
| **28** | Rectus R | 10.959 | 4.4431 | 4.4431 | 0 |
| **29** | Insula L | 0 | 0 | 0 | 0 |
| **30** | Insula R | 0 | 0 | 0 | 0 |
| **31** | Cingulum Ant L | 3.2607 | 0 | 0 | 0 |
| **32** | Cingulum Ant R | 3.3402 | 0 | 0 | 0 |
| **33** | Cingulum Mid L | 0 | 0 | 0 | 0 |
| **34** | Cingulum Mid R | 0 | 0 | 0 | 0 |
| **35** | Cingulum Post L | 18.461 | 8.3048 | 4.4125 | 0 |
| **36** | Cingulum Post R | 20.179 | 3.8201 | 0 | 0 |
| **37** | Hippocampus L | 3.3725 | 0 | 0 | 0 |
| **38** | Hippocampus R | 0 | 0 | 0 | 0 |
| **39** | ParaHippocampal L | 0 | 0 | 0 | 0 |
| **40** | ParaHippocampal R | 0 | 0 | 0 | 0 |
| **41** | Amygdala L | 0 | 0 | 0 | 0 |
| **42** | Amygdala R | 0 | 0 | 0 | 0 |
| **43** | Calcarine L | 14.947 | 4.5207 | 0 | 0 |
| **44** | Calcarine R | 7.7281 | 4.1677 | 4.1677 | 0 |
| **45** | Cuneus L | 16.101 | 9.0764 | 9.0764 | 0 |
| **46** | Cuneus R | 17.777 | 7.7124 | 0 | 0 |
| **47** | Lingual L | 9.857 | 0 | 0 | 0 |
| **48** | Lingual R | 6.5521 | 0 | 0 | 0 |
| **49** | Occipital Sup L | 10.064 | 0 | 0 | 0 |
| **50** | Occipital Sup R | 7.1502 | 3.8056 | 0 | 0 |
| **51** | Occipital Mid L | 0 | 0 | 0 | 0 |
| **52** | Occipital Mid R | 0 | 0 | 0 | 0 |
| **53** | Occipital Inf L | 0 | 0 | 0 | 0 |
| **54** | Occipital Inf R | 10.809 | 3.946 | 0 | 0 |
| **55** | Fusiform L | 0 | 0 | 0 | 0 |
| **56** | Fusiform R | 6.777 | 0 | 0 | 0 |
| **57** | Postcentral L | 0 | 0 | 0 | 0 |
| **58** | Postcentral R | 0 | 0 | 0 | 0 |
| **59** | Parietal Sup L | 0 | 0 | 0 | 0 |
| **60** | Parietal Sup R | 0 | 0 | 0 | 0 |
| **61** | Parietal Inf L | 0 | 0 | 0 | 0 |
| **62** | Parietal Inf R | 0 | 0 | 0 | 0 |
| **63** | SupraMarginal L | 0 | 0 | 0 | 0 |
| **64** | SupraMarginal R | 0 | 0 | 0 | 0 |
| **65** | Angular L | 3.1785 | 0 | 0 | 0 |
| **66** | Angular R | 0 | 0 | 0 | 0 |
| **67** | Precuneus L | 0 | 0 | 0 | 0 |
| **68** | Precuneus R | 0 | 0 | 0 | 0 |
| **69** | Paracentral Lobule L | 0 | 0 | 0 | 0 |
| **70** | Paracentral Lobule R | 0 | 0 | 0 | 0 |
| **71** | Caudate L | 3.2659 | 0 | 0 | 0 |
| **72** | Caudate R | 3.3572 | 0 | 0 | 0 |
| **73** | Putamen L | 3.1799 | 0 | 0 | 0 |
| **74** | Putamen R | 0 | 0 | 0 | 0 |
| **75** | Pallidum L | 0 | 0 | 0 | 0 |
| **76** | Pallidum R | 0 | 0 | 0 | 0 |
| **77** | Thalamus L | 0 | 0 | 0 | 0 |
| **78** | Thalamus R | 0 | 0 | 0 | 0 |
| **79** | Heschl L | 7.6825 | 0 | 0 | 0 |
| **80** | Heschl R | 4.4322 | 0 | 0 | 0 |
| **81** | Temporal Sup L | 0 | 0 | 0 | 0 |
| **82** | Temporal Sup R | 3.3558 | 0 | 0 | 0 |
| **83** | Temporal Pole Sup L | 0 | 0 | 0 | 0 |
| **84** | Temporal Pole Sup R | 0 | 0 | 0 | 0 |
| **85** | Temporal Mid L | 21.58 | 8.103 | 0 | 0 |
| **86** | Temporal Mid R | 7.571 | 0 | 0 | 0 |
| **87** | Temporal Pole Mid L | 0 | 0 | 0 | 0 |
| **88** | Temporal Pole Mid R | 20.737 | 0 | 0 | 0 |
| **89** | Temporal Inf L | 0 | 0 | 0 | 0 |
| **90** | Temporal Inf R | 0 | 0 | 0 | 0 |
| **91** | Cerebelum Crus1 L | 0 | 0 | 0 | 0 |
| **92** | Cerebelum Crus1 R | 0 | 0 | 0 | 0 |
| **93** | Cerebelum Crus2 L | 0 | 0 | 0 | 0 |
| **94** | Cerebelum Crus2 R | 3.1324 | 0 | 0 | 0 |
| **95** | Cerebelum 3 L | 6.5205 | 0 | 0 | 0 |
| **96** | Cerebelum 3 R | 0 | 0 | 0 | 0 |
| **97** | Cerebelum 4 5 L | 0 | 0 | 0 | 0 |
| **98** | Cerebelum 4 5 R | 0 | 0 | 0 | 0 |
| **99** | Cerebelum 6 L | 0 | 0 | 0 | 0 |
| **100** | Cerebelum 6 R | 0 | 0 | 0 | 0 |
| **101** | Cerebelum 7b L | 0 | 0 | 0 | 0 |
| **102** | Cerebelum 7b R | 0 | 0 | 0 | 0 |
| **103** | Cerebelum 8 L | 0 | 0 | 0 | 0 |
| **104** | Cerebelum 8 R | 0 | 0 | 0 | 0 |
| **105** | Cerebelum 9 L | 6.5426 | 0 | 0 | 0 |
| **106** | Cerebelum 9 R | 79.203 | 44.434 | 24.765 | 11.491 |
| **107** | Cerebelum 10 L | 30.634 | 0 | 0 | 0 |
| **108** | Cerebelum 10 R | 20.137 | 0 | 0 | 0 |
| **109** | Vermis 1 2 | 4.561 | 0 | 0 | 0 |
| 110 | Vermis 3 | 0 | 0 | 0 | 0 |
| 111 | Vermis 4 5 | 0 | 0 | 0 | 0 |
| 112 | Vermis 6 | 0 | 0 | 0 | 0 |
| 113 | Vermis 7 | 0 | 0 | 0 | 0 |
| 114 | Vermis 8 | 0 | 0 | 0 | 0 |
| 115 | Vermis 9 | 20.865 | 0 | 0 | 0 |
| 116 | Vermis 10 | 10.39 | 0 | 0 | 0 |
